# A Collocation Method for Numerical Solution of Nonlinear Delay Integro-Differential Equations for Wireless Sensor Network and Internet of Things

**DOI:** 10.3390/s20071962

**Published:** 2020-03-31

**Authors:** Rohul Amin, Shah Nazir, Iván García-Magariño

**Affiliations:** 1 Department of Mathematics, University of Peshawar, Khyber Pakhtunkhwa 25120, Pakistan; raminmath@uop.edu.pk; 2 Department of Computer Science, University of Swabi, Khyber Pakhtunkhwa 23430, Pakistan; shahnazir@uoswabi.edu.pk; 3 Department of Software Engineering and Artificial Intelligence, Complutense University of Madrid, 28040 Madrid, Spain; 4 Instituto de Tecnología del Conocimiento, UCM, 28040 Madrid, Spain

**Keywords:** integro-differential equations, nonlinear delay integro-differential equations, Haar wavelet, internet of things, wireless sensor network

## Abstract

Wireless sensor network and industrial internet of things have been a growing area of research which is exploited in various fields such as smart home, smart industries, smart transportation, and so on. There is a need of a mechanism which can easily tackle the problems of nonlinear delay integro-differential equations for large-scale applications of Internet of Things. In this paper, Haar wavelet collocation technique is developed for the solution of nonlinear delay integro-differential equations for wireless sensor network and industrial Internet of Things. The method is applied to nonlinear delay Volterra, delay Fredholm and delay Volterra–Fredholm integro-differential equations which are based on the use of Haar wavelets. Some examples are given to show the computational efficiency of the proposed technique. The approximate solutions are compared with the exact solution. The maximum absolute and mean square roots errors for distant number of collocation points are also calculated. The results show that Haar method is efficient for solving these equations for industrial Internet of Things. The results are compared with existing methods from the literature. The results exhibit that the method is simple, precise and efficient.

## 1. Introduction

Wireless Sensor Network (WSN) and Industrial Internet of Things (IIoT) have been growing areas of research which are exploited in various fields. These fields include smart home, smart industries, smart transportation, and so on. Nonlinear equation have a vital role in control theory and engineering. Analytical solutions of integral equations (IEs) and integro-differential equations (IDEs), however, either do not exist or are elusive. Several researchers worked in the numerical solution of delay IDEs. The solutions of these equations have a major role in the field of science, particularly in Physics and Engineering. At the point when a physical framework is displayed under the differential sense, it gives an IDE.

This article studies the solution of nonlinear delay Fredholm IDEs (FIDEs), nonlinear delay Volterra IDEs (VIDEs) and nonlinear delay Volterra–Fredholm IDEs (FVIDEs). The nonlinear FIDE with constant delay ξ>0 is:

(1) $$\begin{array}{c} v^{\prime }\left(t\right)=f\left(t,v,\int_{a}^{b}K\left(t,s,v\left(s-\xi \right)\right)ds\right),\\ v(t)=\Phi (t),\;-\xi \leq t<0, \end{array}$$

where the functions *f*, *K*, are sufficiently smooth functions, ξ>0 and v(t) is the unknown function to be determined.

The nonlinear VIDE with constant delay ξ>0 is:

(2) $$\begin{array}{ccc} v^{\prime }\left(t\right) & = & f\left(t,v,\int_{0}^{t}K\left(t,s,v\left(s-\xi \right)\right)ds\right),\\ v(t) & = & \Phi (t),\;-\xi \leq t<0, \end{array}$$

and the nonlinear FVIDE with constant delay ξ>0 is

(3) $$\begin{array}{ccc} v^{\prime }\left(t\right) & = & f\left(t,v,\int_{0}^{t}K_{1}\left(t,s,v\left(s-\xi \right)ds\right),\int_{a}^{b}K_{2}\left(t,s,v\left(s-\xi \right)\right)ds\right),\;\\ v(t) & = & \Phi (t),\;-\xi \leq t<0, \end{array}$$

where the kernels $K_{1}$, $K_{2}$ are smooth functions, *f* is nonlinear function, Φ is the delay condition and v(t) is the unknown function to be determined.The contributions of the proposed research are:

- To develop efficient technique for the approximate solution of nonlinear FVIDEs arising in WSN and IIoT
- To design algorithm for proposed technique
- To examine the efficiency of the developed technique on some test problems and compare the results of our technique with results [1] available in the literature

Many numerical methods have been introduced to solve delay IDEs numerically. But every numerical method has its own shortcomings. In the Haar Wavelets Method (HWM) we focus on improving the efficiency of the direct solvers. The utilization of Haar wavelet has come to noticeable quality amid the most recent two decades. They have far reaching applications in scientific computing, and it is no surprise that they have been extensively used in numerical approximation in the recent literature. Haar wavelet has several applications in diverse areas of research. A portion of the current work utilizing wavelets can be found in the references [2,3,4,5,6,7]. Different researchers utilized wavelets for approximate solutions of IEs [8], boundary value problems [9], PDEs [10], fractional PDEs [11] and delay PDEs [12]. The positive aspects of this method are its simplicity and lesser computation costs: this is due to the sparsity of the transform matrices and to the small number of significant wavelet coefficients. HWM is a computer-oriented method; it gives us the possibility to implement standard subprograms. The HWM is very accurate in detecting singularities of irregular structures and transient phenomena exhibited by the analyzed functions. The reason for using HWM is that it has sparse matrix representation, fast transformation and possibility of implementation of fast and efficient algorithms. The method with far fewer degrees of freedom and with smaller CPU time provides better solutions than classical ones. The negative aspects of HWM are: Haar wavelet uses constant box functions and due to this we need a large number of collocation points in order to achieve better accuracy. This disadvantage can be overcome if Haar wavelet is replaced with some other wavelets having better approximating properties.

The organization of the paper as follows: Haar function is defined in Section 3 and nodal points are also given in this section. In Section 4, numerical technique for nonlinear delay FIDEs, nonlinear delay VIDEs and nonlinear delay FVIDEs arising in WSN and IIoT are given in details. The results are given in tables and graphs in Section 5. Finally, the conclusion is given in Section 7.

## 2. Literature Review

Sometimes the analytical solution of nonlinear delay IDE is difficult to find, then we are interested to find there numerical solution. Several researchers worked on the numerical solution of delay IDEs and developed different methods for solution of these equations. Some of these techniques are Galerkin method [13], Adomian decomposition method [14,15], Haar rationalized functions technique [13], He’s homotopy perturbation technique [16] and variational iteration technique [17]. Brunner [18] used the spline collocation technique to find the solution of Volterra IDEs with unbounded delay. Sadri et al. [19] used Jacobi operational matrices for solution of delay IDEs. In the mentioned method the author converted a given linear and nonlinear equation into a set of linear and nonlinear algebraic equations. By solving these algebraic equations the author found the required solution. Makroglou [20] used block by block technique to found the solution of delay IDEs. Bellour and Bousselsal [21] used collocation method and Taylor polynomials to find the approximate solution of delay IDEs. Rabiei et al. [22], used Runge Kutta method of order five and found the approximate solution of Volterra IDEs. Sekar and Murugesan [23] used Walsh series technique to found the solution of delay Volterra IDEs and system of delay Volterra IDEs. In this technique the authors converted Volterra IDEs and system of delay Volterra IDEs into system of equations. By solving the derived system they obtained the desired solution. Elhawary and Elshami [24] applied a mixed spline and spectral method to find numerical solution of delay Volterra IDEs. Ghomanjani et al. [25] used Bezier curves method to solve Volterra delay IDEs. Sakran [26] used the Chebyshev polynomials method to obtain the solution of first order Volterra IDEs and delay Volterra IDEs. Abubakar and Taiwo [27] used standard integral collocation approximation method to obtain the numerical solution of Fredholm Volterra IDEs. Yuzbasi et al. [28] used Muntz Legendre matrix method to find the approximate solution of linear delay Fredholm IDEs. In this technique the authors convert the given equations into a system of the algebraic equations. Solving this system, the solution is obtained. Khirallah and Mahiub [29] used Galekin’s method with Chabyshev ploynomials to obtain the desired numerical solution. Zaidan [30] obtained the numerical solution of delay IDEs.

## 3. Haar Wavelet

The Haar wavelet (HW) is a piecewise constant function attaining three values 0, 1 and −1. Any function belonging to $L_{2}\left[0,1\right)$, the space of square integrable functions can be approximated utilizing HW function [12]. HW functions form an orthonormal bases for $L_{2}\left[0,1\right)$. Any function v(x) belonging to $L_{2}\left[0,1\right)$ can be written as Haar series: $v\left(x\right)=\sum \lambda_{i}h_{i}\left(x\right)$, where $h_{i}$ are Haar functions [25]. For approximation purposes, this series is truncated at finite terms. Hence, we have the following approximation for the unknown function v(x) that we are trying to approximate.

$$v\left(x\right)\approx \sum_{i=1}^{N}\lambda_{i}h_{i}\left(x\right).$$

Introducing the notation

$$L_{i,1}\left(x\right)=\int_{0}^{x}h_{i}\left(\xi \right)d\xi ,$$

$$L_{i,m+1}\left(x\right)=\int_{0}^{x}L_{i,m}\left(\xi \right)d\xi ,\;\;\;\;m=1,2,3,\cdots$$

These integrals are calculated by above Equation:

(4) $$L_{i,1}\left(x\right)=\left\{\begin{array}{cc} x-\delta_{1} & \mathrm{for}\;x\in [\xi_{1},\xi_{2}),\\ \delta_{3}-x & \mathrm{for}\;x\in [\xi_{2},\xi_{3}),\\ 0 & \mathrm{elsewhere}. \end{array}\right.$$

Let $I=[\xi_{1},\xi_{2}]$ be an interval on which the given delay IDE is to be solved on interval *I*, then *I* is divided into subintervals by formula:

(5) $$t_{k}=\xi_{1}+\left(\xi_{2}-\xi_{1}\right)\frac{k-1/2}{2M},\;k=1,2,3,\cdots ,2M.$$

where 2M=N is natural number. The points $t_{k}$ are known as collocation points (CP) or nodal points.

This paper answers the following research questions in brief:

- To find solutions of different nonlinear FVIDEs arising in WSN and IIoT
- To check the efficiency and accuracy of the developed technique, the proposed method is applied on some test problems

## 4. Numerical Method for Nonlinear Delay IDEs Arising in WSN and IIoT

The current approach proposes an efficient numerical method for WSN and IIoT using HWM for nonlinear delay FIDEs (1), nonlinear delay VIDEs (2) and nonlinear delay FVIDEs (3). In the first subsection, nonlinear delay FIDEs will be studied. In next subsection nonlinear delay VIDEs will be considered. Nonlinear delay FVIDEs will be discussed in the third subsection.

Introduce some notations: $\Theta =\sum_{i=1}^{N}$. Let $v^{\prime }\left(t\right)$ in $L_{2}\left[0,1\right)$, then

(6) $$v^{\prime }\left(t\right)=\Theta \lambda_{i}h_{i}\left(t\right).$$

Integrating, we have

(7) $$v\left(t\right)=v_{0}+\Theta \lambda_{i}L_{i,1}\left(t\right),$$

where $v\left(0\right)=v_{0}$. The integrals in the above Equations (1)–(3) are calculated by following Haar integral formula:

(8) $$\int_{\xi_{1}}^{\xi_{2}}g\left(t\right)dt\approx \frac{\xi_{2}-\xi_{1}}{N}\Theta g\left(t_{k}\right)=\Theta f\left(\xi_{1}+\frac{\left(\xi_{2}-\xi_{1}\right)\left(k-1/2\right)}{N}\right).$$

By putting these expressions and nodal points from Equation (5) in a given nonlinear delay FIDEs to obtain system of nonlinear equations. The unknown coefficients $\lambda_{i}$’s, are obtained by solving this system. The above system is solved by Broyden’s technique.

### 4.1. Nonlinear Delay Fredholm IDEs

In this section, we will describe numerical technique to solve nonlinear delay FID Equation (1). Applying HW and above formula to Equation (1), we get

$$\Theta \lambda_{i}h_{i}\left(t\right)=f\left(t,v_{0}+\Theta \lambda_{i}L_{i,1}\left(t\right),\int_{a}^{b}K\left(t,s,\Phi \left(s-\xi \right)\right)ds\right),$$

by simplification, we have

$$\Theta \lambda_{i}h_{i}\left(t\right)-f\left(t,v_{0}+\Theta \lambda_{i}L_{i,1}\left(t\right),\int_{a}^{b}K\left(t,s,\Phi \left(s-\xi \right)\right)ds\right)=0,$$

putting the nodal points (5), we get the following nonlinear system

$$\Theta \lambda_{i}h_{i}\left(t_{j}\right)-f\left(t_{j},v_{0}+\Theta \lambda_{i}L_{i,1}\left(t_{j}\right),\int_{a}^{b}K\left(t_{j},s,\Phi \left(s-\xi \right)\right)ds\right)=0.$$

$$\mathrm{Let}\;\;F_{j}=\Theta \lambda_{i}h_{i}\left(t_{j}\right)-f\left(t_{j},v_{0}+\Theta \lambda_{i}p_{i,1}\left(t_{j}\right),\int_{a}^{b}K\left(t_{j},s,\Phi \left(s-\xi \right)\right)ds\right),$$

where the integral is calculated analytically by using Equation (8). The above system can be written as

$$\mathrm{Let}\;\;F_{j}=\Theta \lambda_{i}h_{i}\left(t_{j}\right)--f\left(t_{j},v_{0}+\Theta \lambda_{i}L_{i,1}\left(t_{j}\right),\frac{b-a}{N}\sum_{m=1}^{N}K\left(t_{j},s_{m},\Phi \left(s_{m}-\xi \right)\right)\right).$$

The above system is solved by Broyden technique [31]. The Jacobian is given by

(9) $$J={\left[J_{jk}\right]}_{N\times N},$$

where

(10) $$J_{jk}=\frac{\partial F_{j}}{\partial a_{k}}=h_{k}\left(t_{j}\right)-\frac{\partial f}{\partial \lambda_{k}}L_{k,1}\left(t_{j}\right).$$

The solution of this system gives the values of the unknown coefficients $\lambda_{i}^{\prime }s$. The solution v(t) at nodal points is calculated by putting $\lambda_{i}{,}^{\prime }s$ in Equation (6).

### 4.2. Nonlinear Delay Volterra IDEs

In this section HWC technique is developed for solution of nonlinear delay VID Equation (2). Applying the HW approximations to the above Equation (2) and using the delay condition, we obtain

$$\Theta \lambda_{i}h_{i}\left(t\right)=f\left(t,v_{0}+\Theta \lambda_{i}p_{i,1}\left(t\right),\int_{0}^{t}K\left(t,s,\Phi \left(s-\xi \right)\right)ds\right),$$

by simplification, we obtain

$$\Theta \lambda_{i}h_{i}\left(t\right)-f\left(t,v_{0}+\Theta \lambda_{i}L_{i,1}\left(t\right),\int_{0}^{t}K\left(t,s,\Phi \left(s-\xi \right)\right)ds\right)=0,$$

putting the nodal points (5), we obtain the following nonlinear system

$$\Theta \lambda_{i}h_{i}\left(t_{j}\right)-f\left(t_{j},v_{0}+\sum_{i=1}^{N}\lambda_{i}L_{i,1}\left(t_{j}\right),\int_{0}^{t}K\left(t_{j},s,\Phi \left(s-\xi \right)\right)ds\right)=0.$$

$$\Theta \lambda_{i}h_{i}\left(t_{j}\right)-f\left(t_{j},v_{0}+\sum_{i=1}^{N}\lambda_{i}L_{i,1}\left(t_{j}\right),\int_{0}^{t}K\left(t_{j},s,\Phi \left(s-\xi \right)\right)ds\right)=0.$$

$$\mathrm{Let}\;\;F_{j}=\Theta \lambda_{i}h_{i}\left(t_{j}\right)-f\left(t_{j},v_{0}+\Theta \lambda_{i}L_{i,1}\left(t_{j}\right),\int_{a}^{b}K\left(t_{j},s,\Phi \left(s-\xi \right)\right)ds\right),$$

applying the HW integration formula (8). The above system can be written as

$$\mathrm{Let}\;\;F_{j}=\Theta \lambda_{i}h_{i}\left(t_{j}\right)-f\left(t_{j},u_{0}+\Theta \lambda_{i}L_{i,1}\left(t_{j}\right),\frac{t}{N}\Theta K\left(t_{j},s_{m},\Phi \left(s_{m}-\xi \right)\right)\right).$$

The above system is solved by Broyden technique [31]. The Jacobian is given by

(11) $$J={\left[J_{jk}\right]}_{N\times N},$$

where

(12) $$J_{jk}=\frac{\partial F_{j}}{\partial a_{k}}=h_{k}\left(t_{j}\right)-\frac{\partial f}{\partial \lambda_{k}}L_{k,1}\left(t_{j}\right),$$

The solution of this system gives values of $\lambda_{i}^{\prime }s$. The solution v(t) at nodal points is calculated by putting $\lambda_{i}^{\prime }s$ in Equation (6).

### 4.3. Nonlinear Delay Volterra–Fredholm IDEs

A HWC technique is developed for the solution of nonlinear delay FVID Equation (3) in this section. Applying the Haar wavelet approximations to the above Equation (3) and using delay condition, we obtain

$$\Theta \lambda_{i}h_{i}\left(t\right)=f\left(t,v_{0}+\Theta \lambda_{i}L_{i,1}\left(t\right),\int_{0}^{t}K_{1}\left(t,s,\Phi \left(s-\xi \right)ds\right),\int_{a}^{b}K_{2}\left(t,s,\Phi \left(s-\xi \right)\right)ds\right),$$

by simplification, we have

$$\Theta \lambda_{i}h_{i}\left(t\right)-f\left(t,v_{0}+\sum_{i=1}^{N}\lambda_{i}L_{i,1}\left(t\right),\int_{0}^{t}K_{1}\left(t,s,\Phi \left(s-\xi \right)ds\right),\int_{a}^{b}K_{2}\left(t,s,\Phi \left(s-\xi \right)\right)ds\right)=0,$$

putting the nodal points (5), we obtain a system of nonlinear equations given below

$$\Theta \lambda_{i}h_{i}\left(t_{j}\right)-f\left(t_{j},v_{0}+\Theta \lambda_{i}p_{i,1}\left(t_{j}\right),\int_{0}^{t}K_{1}\left(t_{j},s,\Phi \left(s-\xi \right)ds\right),\int_{a}^{b}K_{2}\left(t_{j},s,\Phi \left(s-\xi \right)\right)ds\right)=0.$$

$$\mathrm{Let}\;F_{j}=\Theta \lambda_{i}h_{i}\left(t_{j}\right)-f\left(t_{j},v_{0}+\Theta \lambda_{i}p_{i,1}\left(t_{j}\right),\int_{0}^{t}K_{1}\left(t_{j},s,\Phi \left(s-\xi \right)ds\right),\int_{a}^{b}K_{2}\left(t_{j},s,\Phi \left(s-\xi \right)\right)ds\right),$$

applying the Haar wavelet integration formula (8). The above system become

$$\begin{array}{cc} \mathrm{Let}\; & F_{j}=\Theta \lambda_{i}h_{i}\left(t_{j}\right)-f\left(t_{j},v_{0}+\Theta \lambda_{i}L_{i,1}\left(t_{j}\right),\right.\\ & \left.\frac{t}{N}\Theta K_{1}\left(t_{j},s_{m},\Phi \left(s_{m}-\xi \right)\right),\frac{b-a}{N}\Theta K_{2}\left(t_{j},s_{m},\Phi \left(s_{m}-\xi \right)\right)\right). \end{array}$$

The above system is solved by Broyden technique [31]. The Jacobian is given by

(13) $$J={\left[J_{jk}\right]}_{N\times N},$$

where

(14) $$J_{jk}=\frac{\partial F_{j}}{\partial a_{k}}=h_{k}\left(t_{j}\right)-\frac{\partial f}{\partial \lambda_{k}}L_{k,1}\left(t_{j}\right),$$

The solution of this system gives the values of the unknown Haar coefficients $\lambda_{i}^{\prime }s$. The required solution v(t) at grid points is calculated by substituting $\lambda_{i}^{\prime }s$ in Equation (6).

## 5. Numerical Assessments

In this section, we demonstrate the capability of the introduced approach. The proposed HWC technique is used for solving some examples. To show the efficiency of the method, the numerical solutions are compared with the exact solutions and it is presented for each examples in tables and figures. The notation $L_{abs}$ is used for maximum absolute error and $M_{c}\left(N\right)$ is used for mean square root errors at *N* collocation points. If $\hat{v}$ denotes the approximate solution and *v* denotes the exact solution at *N* nodal points, then $M_{c}\left(N\right)$ is given by:

(15) $$M_{c}\left(N\right)=\sqrt{\frac{1}{N}\Theta {\left(v-\hat{v}\right)}^{2}}.$$

Test Problem 1. Consider the nonlinear delay FIDE [1]

(16) $$\begin{array}{ccc} v^{\prime }\left(t\right) & = & -v\left(t\right)+\int_{0}^{1}v^{2}\left(\frac{s}{2}\right)ds-e^{-1}+1,\\ v(t) & = & e^{-t},\;\;t\leq 0. \end{array}$$

The exact solution is given by

(17) $$v\left(t\right)=e^{-t}.$$

**Figure 1:**
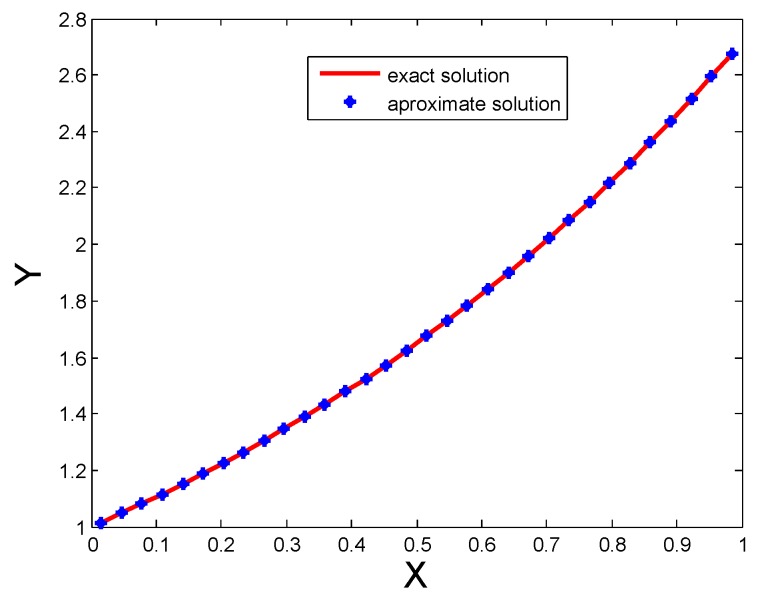
Comparison of both solutions for N=32 for Test Problem 1.

The $L_{abc}$ and $M_{c}\left(N\right)$ errors for distinct numbers of CPs are shown in Table 1. The solution obtained using HWCM is good as shown in table. It is evident that both $L_{abc}$ and $M_{c}\left(N\right)$ errors are decreased by increasing the number of CPs. From this table it is clear that the proposed technique is better than spline functions technique [1]. Moreover, we see that both the $L_{abc}$ and $M_{c}\left(N\right)$ errors are decreased up to order $10^{-6}$ for M=256 CPs. The comparison of both solutions for N=32 are also shown in Figure 1.

Test Problem 2. Consider the following nonlinear delay VIDE [1]

(18) $$\begin{array}{c} v^{\prime }\left(t\right)=v^{2}\left(\frac{t}{2}\right)+\int_{0}^{t}v^{2}\left(\frac{s}{2}\right)ds-e^{t}+1,\\ v\left(t\right)=e^{t},\;\;t\leq 0. \end{array}$$

The exact solution is given by

(19) $$v\left(t\right)=e^{t}.$$

**Figure 2:**
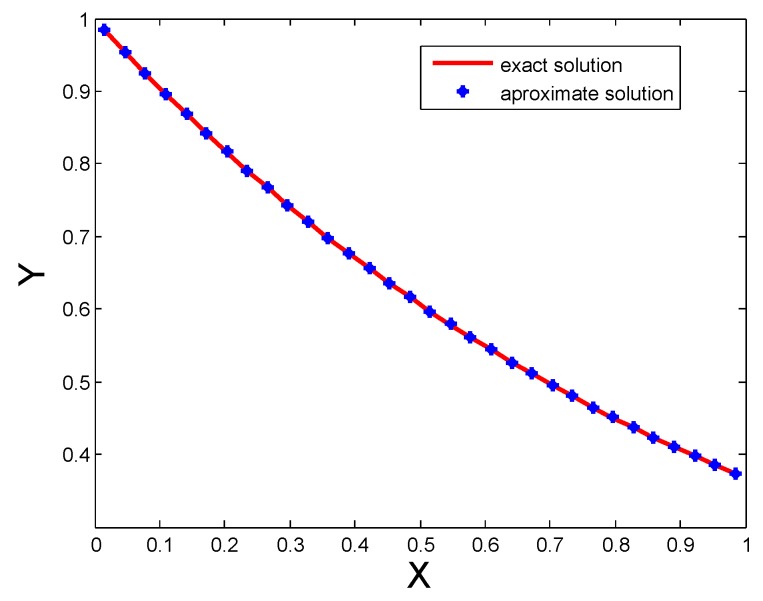
Comparison of both solutions for N=32 for Test Problem 2.

In Table 2, $L_{abc}$ and $M_{c}\left(N\right)$ errors for different numbers of CPs are shown, the better performance in terms of accuracy can be observed from this table. The numerical results are also shown in Figure 2. The figure shows that for a small number of CPs the performance of the proposed method is good, also we see that if we increase the number of CPs the accuracy gets betters. From the table it is clear that the performance of the proposed technique is better than Spline functions [1].

Test Problem 3. Consider the following nonlinear delay FVIDE

(20) $$\begin{array}{c} v^{\prime }\left(t\right)=g\left(t\right)+\int_{0}^{t}\left(t-x\right)v^{2}\left(\frac{x}{2}\right)dx+\int_{0}^{1}\left(t+x\right)v\left(x\right)dx,\\ v\left(t\right)=t^{2}-2,\;\;t\leq 0. \end{array}$$

The function f(t) is chosen such that the exact solution is

(21) $$y\left(t\right)=t^{2}-2.$$

**Figure 3:**
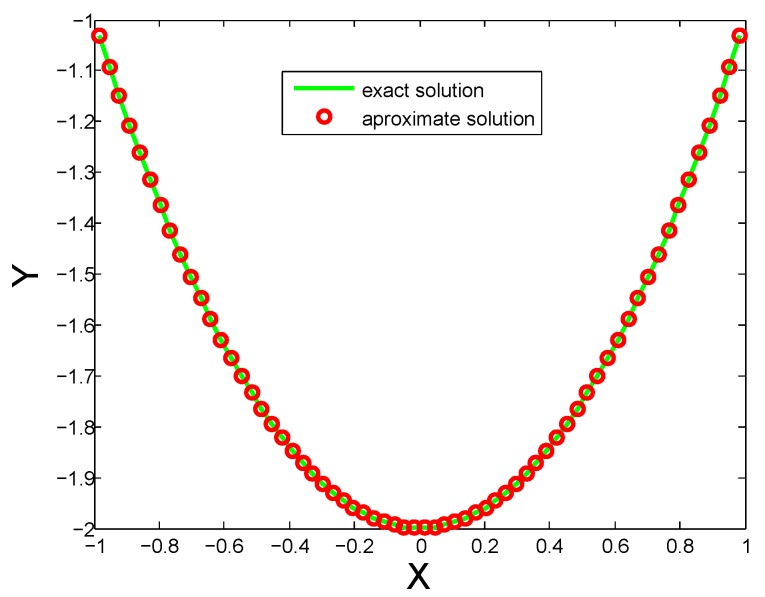
Comparison of both solutions for N=32 for Test Problem 3.

This example is solved numerically by using the proposed HWC technique. Accuracy up to 9 decimal places is reported in Table 3 corresponding to 1024 CPs. It can be observed from the table that the accuracy is increased by increasing the number of CPs. The comparison of exact and approximate solutions are also shown in Figure 3.

## 6. Results and Discussion

Numerical approximation methods are usually needed for solution of equations when the equations are nonlinear. The Haar wavelet can be implemented on problems which have exact solutions. After successful implementation, we analyzed that if the error is significant, then we apply it to our proposed governing equations nonlinear delay FIDEs, VIDEs and FVIDEs. $L_{abc}$ and $M_{c}\left(N\right)$ errors for distinct numbers of CPs are shown in tables and graphs.

Table 1 and Table 2 show $L_{abc}$, $M_{c}\left(N\right)$ errors and results of [1] for distinct numbers of CPs for Test Problem 1 and Test Problem 2. It is evident that both $L_{abc}$ and $M_{c}\left(N\right)$ errors are decreased by increasing the number of CPs. Comparison of both exact and approximate solutions for 32 CPs are shown in Figure 1 for Test Problem 1, Figure 2 for Test Problem 2 and Figure 3 for Test Problem 3.

The proposed HWM numerical technique can be applied for the solution of a system of nonlinear delay IDEs. Also, this method can be applied for higher order nonlinear delay FIDEs, VIDEs and FVIDEs.

## 7. Conclusions

WSNs and IoT represent a research area of up-growing interest, which is exploited in various fields such as smart home, smart industries, smart transportation, and so on. HWCM is developed for numerical solution of nonlinear delay IDEs, i.e., we have considered nonlinear delay FIDEs, VIDEs and FVIDEs, and studied HWCM for solution of these equations. The delay IDEs are converted into a system of nonlinear algebraic equations, then this system is solved by the Broyden technique. Some test problems are presented showing that the proposed technique can converge with a good speed. From these test problems, we conclude that this approach can obtain very accurate and satisfactory results. A good performance of HWCM is also observed when the method is tested on some problems of these equations from literature. However, for more accuracy we need more CPs, which is the only limitation of the proposed technique because selection of a large number of CPs result in increased computational cost due to inversion of the Haar matrix. All algorithms of the proposed technique are implemented in MATLAB and Mathematica software.

## Figures and Tables

**Table 1:** $L_{abc}$ and $M_{c}\left(N\right)$ errors for Test Problem 1.

J	$N=2^{J+1}$	$L_{abc}$	$M_{c}\left(N\right)$	Results [1]
1	4	2.1194 $\times 10^{-5}$	1.5933 $\times 10^{-5}$	4.84 $\times 10^{-3}$
2	8	6.3911 $\times 10^{-6}$	4.0412 $\times 10^{-6}$	1.36 $\times 10^{-2}$
3	16	1.7632 $\times 10^{-6}$	1.0141 $\times 10^{-6}$	2.01 $\times 10^{-2}$
4	32	4.6373 $\times 10^{-7}$	2.5447 $\times 10^{-7}$	3.54 $\times 10^{-2}$
5	64	1.1898 $\times 10^{-7}$	6.4159 $\times 10^{-8}$	3.85 $\times 10^{-2}$
6	128	3.0129 $\times 10^{-8}$	1.6638 $\times 10^{-8}$	—
7	256	7.5791 $\times 10^{-9}$	4.9327 $\times 10^{-9}$	—

**Table 2:** $L_{abc}$ and $M_{c}\left(N\right)$ errors for Test Problem 2.

J	$N=2^{J+1}$	$L_{abc}$	$M_{c}\left(N\right)$	Results [1]
1	4	5.4262 $\times 10^{-4}$	4.6432 $\times 10^{-4}$	1.1 $\times 10^{-1}$
2	8	1.5086 $\times 10^{-4}$	3.1776 $\times 10^{-5}$	1.1 $\times 10^{-2}$
3	16	3.9748 $\times 10^{-5}$	2.9549 $\times 10^{-5}$	1.9 $\times 10^{-2}$
4	32	1.0203 $\times 10^{-5}$	7.3941 $\times 10^{-6}$	2.8 $\times 10^{-2}$
5	64	2.5847 $\times 10^{-6}$	1.8489 $\times 10^{-6}$	9.5 $\times 10^{-4}$
6	128	6.5045 $\times 10^{-7}$	4.6226 $\times 10^{-7}$	—
7	256	1.6315 $\times 10^{-7}$	1.1556 $\times 10^{-7}$	—
8	512	4.0855 $\times 10^{-8}$	2.8892 $\times 10^{-8}$	—
9	1024	1.0222 $\times 10^{-8}$	7.2230 $\times 10^{-9}$	—
10	2048	2.5566 $\times 10^{-9}$	1.8057 $\times 10^{-9}$	—

**Table 3:** $L_{abc}$ and $M_{c}\left(N\right)$ errors for Test Problem 3.

J	$N=2^{J+1}$	$L_{abc}$	$M_{c}\left(N\right)$
1	4	4.71362 $\times 10^{-4}$	2.89263 $\times 10^{-4}$
2	8	1.02141 $\times 10^{-4}$	1.00189 $\times 10^{-4}$
3	16	3.72674 $\times 10^{-5}$	3.60812 $\times 10^{-5}$
4	32	2.61325 $\times 10^{-5}$	1.46973 $\times 10^{-5}$
5	64	4.58470 $\times 10^{-6}$	2.25831 $\times 10^{-6}$
6	128	1.84356 $\times 10^{-6}$	3.96374 $\times 10^{-7}$
7	256	4.01407 $\times 10^{-7}$	1.32281 $\times 10^{-7}$
8	512	1.37851 $\times 10^{-7}$	2.13459 $\times 10^{-8}$
9	1024	3.93057 $\times 10^{-8}$	4.53722 $\times 10^{-9}$
